# Use of Patient-Reported Outcome Measures in Clinical Studies of Chronic Myeloid Leukemia: A Scoping Literature Review

**DOI:** 10.1007/s11899-025-00755-0

**Published:** 2025-10-15

**Authors:** Kathryn E. Flynn, Lovneet Saini, Aditi Kataria, Kejal Jadhav, Daisy Yang, David Wei

**Affiliations:** 1 Department of Medicine, Division of Hematology and Oncology, Medical College of Wisconsin, Milwaukee, WI USA; 2 Novartis Healthcare Pvt. Ltd, Hyderabad, India; 3 Novartis Pharmaceuticals Corporation, East Hanover, NJ USA

**Keywords:** Chronic Myeloid Leukemia, Patient-Reported Outcome Measures, Health-Related Quality of Life, Tyrosine Kinase Inhibitor

## Abstract

**Purpose of Review:**

Management of chronic myeloid leukemia (CML) with tyrosine kinase inhibitors has improved patient survival. However, patient quality of life (QOL) continues to be impacted by disease symptoms and treatment-related adverse events. Patient-reported outcome measures (PROMs) provide evidence of the patient experience. A scoping literature review was conducted to identify and summarize the evidence on PROMs used for patients with CML.

**Recent Findings:**

Embase and MEDLINE databases were searched for publications from 2001 to 2023 that reported PROMs. Ongoing and completed trials listed on ClinicalTrials.gov were also reviewed. Results were summarized according to the PROMs used and the information collected in these PROMs. After screening 6337 records, 208 unique studies were identified with published PRO evidence reporting data from 92 unique PROMs. The most commonly reported PROMs (in ≥5% of publications) were used in 115 studies, of which 45 were exclusively in the frontline setting. The most commonly used PROMs in studies in the frontline setting were variations of European Organisation for Research and Treatment of Cancer QLQ, Functional Assessment of Chronic Illness Therapy Measurement System/Functional Assessment of Cancer Therapy, and 36-Item Short Form Survey.

**Summary:**

This scoping literature review highlighted that a variety of PROMs are used in CML studies, including studies in the frontline setting. Different QOL aspects are measured by commonly used PROMs, and the choice of PROM is dependent on the study setting and objectives. A more comprehensive understanding of QOL gained by using appropriate PROMs will help optimize patient-centered treatment selection in CML.

**Supplementary Information:**

The online version contains supplementary material available at 10.1007/s11899-025-00755-0.

## Introduction

Chronic myeloid leukemia (CML) is a slowly progressing disease of the blood and bone marrow, usually occurring during and after middle age [1–3]. In 2022, an estimated 74,198 people were living with CML in the US, and > 9560 new cases are estimated for 2025 [4]. The development of tyrosine kinase inhibitors (TKIs) transformed the treatment paradigm for CML. Among TKI users, life expectancy approaches that of the general population [5, 6], although worse survival is reported in patients with CML in countries, including the US, that do not have universal health insurance, which restricts patient access to TKIs [7]. Moreover, as patients with CML live longer on treatments that are usually lifelong, even low-grade adverse events (AEs) from TKIs can significantly impact daily living and become a critical aspect of survivorship [8, 9]. While the advent of several TKIs for CML treatment offers patients more treatment options, it is important to understand the different AE profiles of these TKIs and how they impact quality of life (QOL) [9].

Patient-reported outcomes (PROs) are direct reports from patients about their QOL, functional status, symptoms, health-related behaviors, and satisfaction with care, without interpretation by clinicians or others [10, 11]. PRO measures (PROMs) are tools used to capture this information in clinical research, providing critical insight into treatment effects from the patient perspective. By reflecting concepts that are meaningful to patients, they can be used to complement clinical outcomes, such as biomarkers and disease response rates [12]. These tools can be “generic” (that is, not disease specific) and fit a variety of different conditions, while others are disease specific [12, 13]. The use of PROMs is recognized for its potential to transform health care through patient-centered management [14] and improve clinician-patient communication to guide decision-making [10, 14] that can ultimately improve QOL [15]. However, selecting appropriate PROMs requires consideration of multiple methodological aspects [13, 16]. Selected PROMs should help manage individual patient care and support broader system needs (e.g., performance evaluation, healthcare planning, and policy-making) [13]. PROMs should be valid, reliable, and responsive to ensure the quality of the information being collected [16]. Feasibility, timing and frequency, and interpretability of PROMs should also be considered [13]. Often, the choice of PROM is based on professional judgment [12], and, with a wide variety of generic and disease-specific options [12, 13], establishing a consensus for which PROM to use in clinical trials can be challenging [12, 16].

To describe the existing landscape for PROM usage in patients with CML, we conducted a scoping literature review to identify and summarize the evidence from published studies and ongoing trials, including clinical trials and observational studies, on PROMs used to evaluate QOL, particularly in the frontline setting and US-based studies.

## Methods

### Search Strategy and Data Collection Process

A scoping literature review was conducted in accordance with PRISMA guidelines [17]. Embase and MEDLINE databases were searched using the Embase platform. Search queries included disease terms, PRO and QOL terms, and study design terms outlined in **Tables S1** and **S2**. These searches were supplemented by a search of ClinicalTrials.gov for ongoing and completed phase 2 and 3 trials in patients with CML and a bibliographic search of review articles.

### Study Selection

The search focused on articles published between January 2001 (when imatinib was approved [18]) and September 2023 that reported PROMs in adult patients diagnosed with CML irrespective of line of therapy and disease phase. Clinical trials, observational studies, real-world evidence studies, and systematic literature reviews and meta-analyses were included, regardless of interventions or comparators implemented. Animal and in vitro studies; reviews, editorials, notes, and opinions; case studies and reports; and case series were excluded.

### Data Extraction

The retrieved publications were screened in a 2-step process, first based on title and abstract, and then by full-text screening. The second screening grouped multiple publications of a single study to avoid repetition. Screening was conducted by 2 independent reviewers, and a third reviewer adjudicated any discrepancies. A list of CML studies reporting the use of PRO instruments was compiled. The list was refined based on predefined inclusion and exclusion criteria (Table 1). Studies were stratified for US-based studies and studies in the frontline setting at screening. A list of PROMs used in these studies was compiled, and the most commonly reported PROMs, defined as those appearing in ≥ 5% of publications, were extracted into a predefined MS Excel–based grid. Data on any other PROMs reported in these studies were also extracted. After the scoping literature review was conducted, details about these PROMs were gathered to compare key characteristics of each measure, including type of PRO (generic, oncology specific, or CML specific), target population, content, recall period, number of items, time to completion, and available languages [19].

**Table 1:** Study criteria

Inclusion criteria	Exclusion criteria
***Patient population***	
• Adult patients (aged ≥ 18 years) diagnosed with CML (irrespective of line of therapy and disease phase)	• Diseases other than CML • Animal/in vitro studies
***Intervention/comparators***	
• NA	• No exclusion based on intervention/comparator
***Studies reporting the following outcomes***^***a***^	
• PROs assessing quality of life of patients • PROs assessing functional endpoints (i.e. functional PROs) • PROs assessing clinical outcomes • PROs looking at specific symptom burden (e.g. fatigue or pain) • PROs assessing caregiver burden • PROs for medication adherence	• Outcomes other than reported
***Study design***	
• Clinical trials • Observational studies • Real-world evidence • Systematic literature reviews, meta-analyses	• Reviews, editorials, notes, opinions • Case studies, case reports • Case series
***Language***	
• English	• Non-English
***Publication date***	
• NA	• NA
***Country***	
• No limits	• NA

CML, chronic myeloid leukemia; NA, not applicable; PRO, patient-reported outcome. ^a^ Please note that this is not an exhaustive list

## Results

### Study Characteristics

**Fig. 1:**
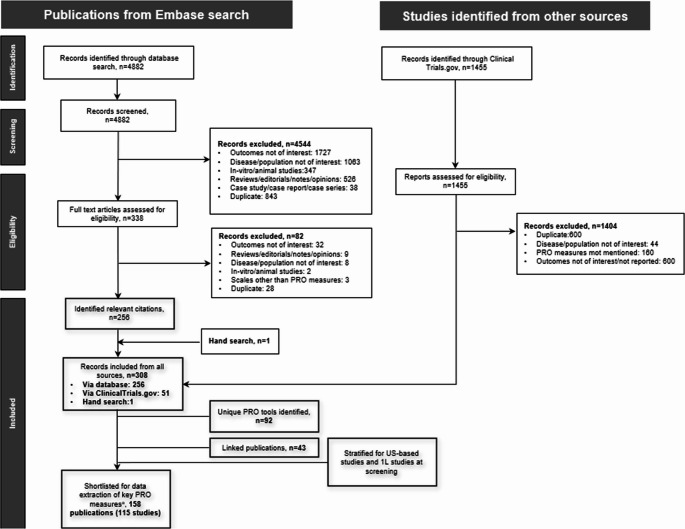
PRISMA flow diagram illustrating the study selection process for the scoping literature review. 1L, first line; PRISMA, Preferred Reporting Items for Systematic reviews and Meta-Analyses; PRO, patient-reported outcome. ^a ^Data were extracted for the PRO measures used in ≥5% of studies

In total, 6337 records were retrieved from Embase, MEDLINE, and ClinicalTrials.gov (Fig. 1). Of these, 4882 records were from Embase, and 1455 were from ClinicalTrials.gov. Further screening based on exclusion criteria, duplications, and irrelevant citations identified 308 relevant CML publications with PROMs from all sources (database searches, *n* = 256; ClinicalTrials.gov, *n* = 51; hand search, *n* = 1). Of these, 256 records had published PRO evidence. Multiple records from single studies were linked, and the list of publications was pared down to 208 unique studies with PRO evidence. Of these 208 studies, 84 were conducted in Europe, 43 in Asia, 40 in North America, and 19 in multiple countries globally. The remaining 22 studies were conducted in Africa, Middle East, Oceania, and South America. Fifty-four of the 208 studies had ≥ 1 study center in the US, and 36 were entirely based in the US. Eighty-one of the 208 studies included patients with CML in the frontline setting or beyond, and 45 exclusively included patients in the frontline setting.

The most commonly reported PROMs (in ≥ 5% of publications) were used in 115 of the 208 unique studies, which included 82 observational studies, 29 clinical trials, 3 prospective cohort studies, and 1 secondary analysis of combined data. Of these 115 studies, 22 were conducted in multiple centers in multiple countries, of which 16 were across continents and considered global. Forty-one of the 115 studies were conducted in ≥ 1 European country, 33 had a center in the US, and 16 were entirely based in the US. Sixty-one of the 115 studies included patients in the frontline setting, and 40 of these studies exclusively involved patients in the frontline setting. The most common intervention in these 115 studies was imatinib (*n* = 56), followed by nilotinib (*n* = 37), dasatinib (*n* = 31), bosutinib (*n* = 16), interferon (*n* = 6), ponatinib (*n* = 5), asciminib (*n* = 5), and hydroxyurea (*n* = 1). The median age of patients, reported in 53 of the 115 studies, ranged from 36.0 to 72.5 years, with > 50% male patients (range, 19%−78%). The sample size ranged from 11 to 2546 patients. Of the 115 studies, 57 evaluated patients with CML in chronic phase, 10 had a mix of patients with chronic- and acute-phase CML, and 48 did not report the phase.

### Analysis

**Fig. 2:**
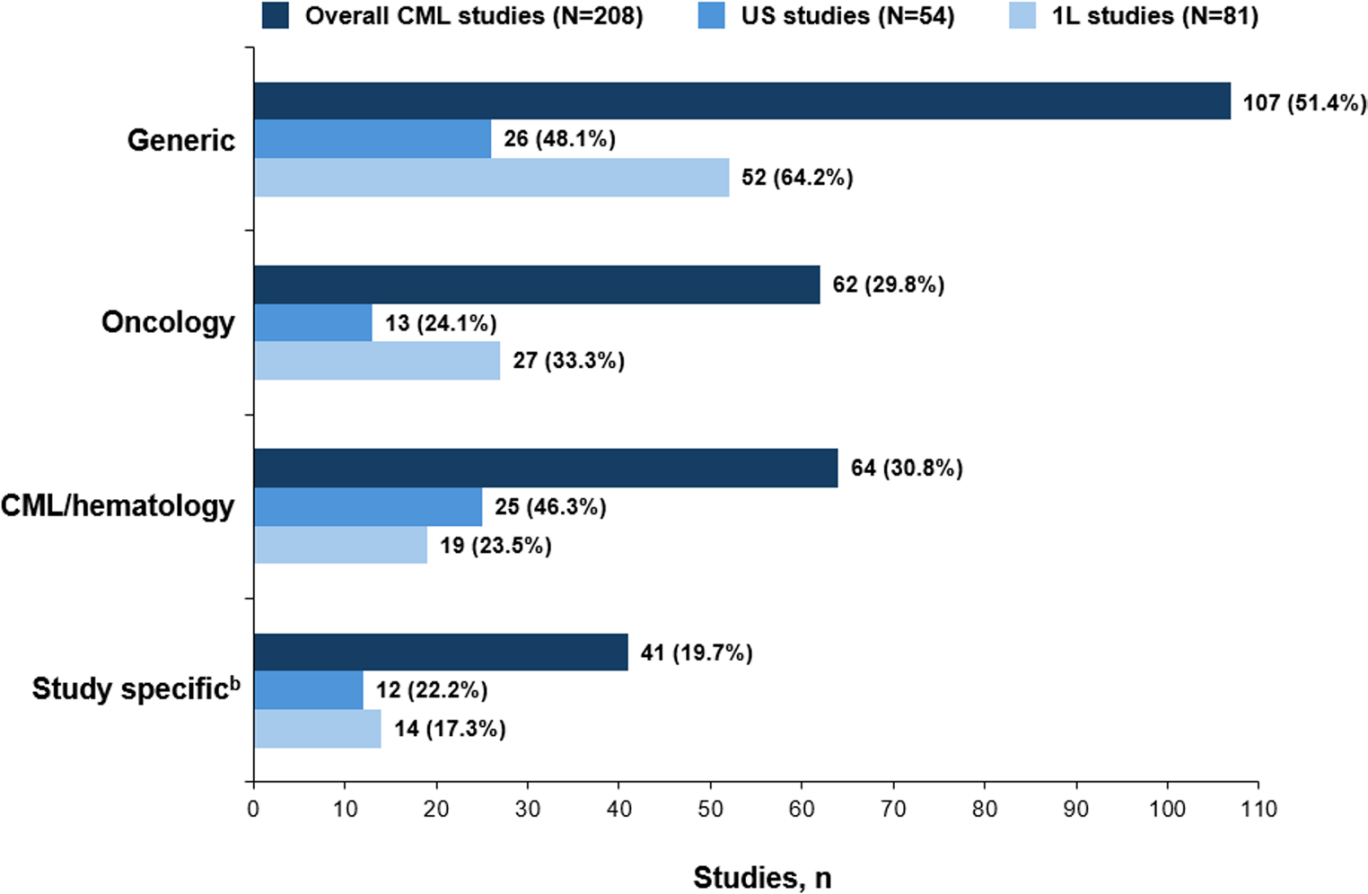
PROMs identified across the studies.^a ^1L, first line; CML, chronic myeloid leukemia; PROM, patient-reported outcome measure. ^a^ Numbers are not mutually exclusive as some studies report multiple measures. ^b^ If the PROM was not named or a nonvalidated questionnaire/interview was used, it was categorized as study specific

The 208 unique studies with published PRO evidence reported data from 92 unique PROMs, including 62 generic, 19 oncology specific, and 11 CML/leukemia/hematology specific. PROMs that were not named or validated were categorized as study specific and counted as 1 of the 12 CML/leukemia/hematology-specific PROMs. Generic, oncology-specific, CML/leukemia/hematology-specific, and study-specific PROMs, respectively, were used in 51.4%, 29.8%, 30.8%, and 19.7% of all 208 CML studies; 48.1%, 24.1%, 46.3%, and 22.2% of the 54 US studies; and 64.2%, 33.3%, 23.5%, and 17.3% of the 81 CML studies in the frontline setting (Fig. 2).

Of the 92 unique PROMs, generic PROMs that were reported in ≥ 5 publications included the 36-Item Short Form Survey (SF-36), European Quality of Life 5 Dimensions (EQ-5D), Morisky Medication Adherence Scale (MMAS, MMAS-8, and MMAS-4), Functional Assessment of Chronic Illness Therapy Measurement System (FACIT), FACIT-Fatigue (FACIT-F), Patient-Reported Outcomes Measurement Information System (PROMIS), Hospital Anxiety and Depression Scale (HADS), and Patient Health Questionnaire-9 (PHQ-9). Oncology-specific PROMs reported in ≥ 5 publications included the European Organisation for Research and Treatment of Cancer (EORTC) Quality of Life Questionnaire Core-30 (QLQ-C30) and Functional Assessment of Cancer Therapy-Biologic Response Modifiers (FACT-BRM). CML/leukemia/hematology-specific PROMs reported in ≥ 5 publications included the EORTC QLQ-CML24, Functional Assessment of Cancer Therapy-Leukemia (FACT-Leu), MD Anderson Symptom Inventory-CML (MDASI-CML), Haematological Malignancy Patient Reported Outcome (HM-PRO), and study-specific questionnaires with no specific scale mentioned (nonvalidated tools).

**Fig. 3:**
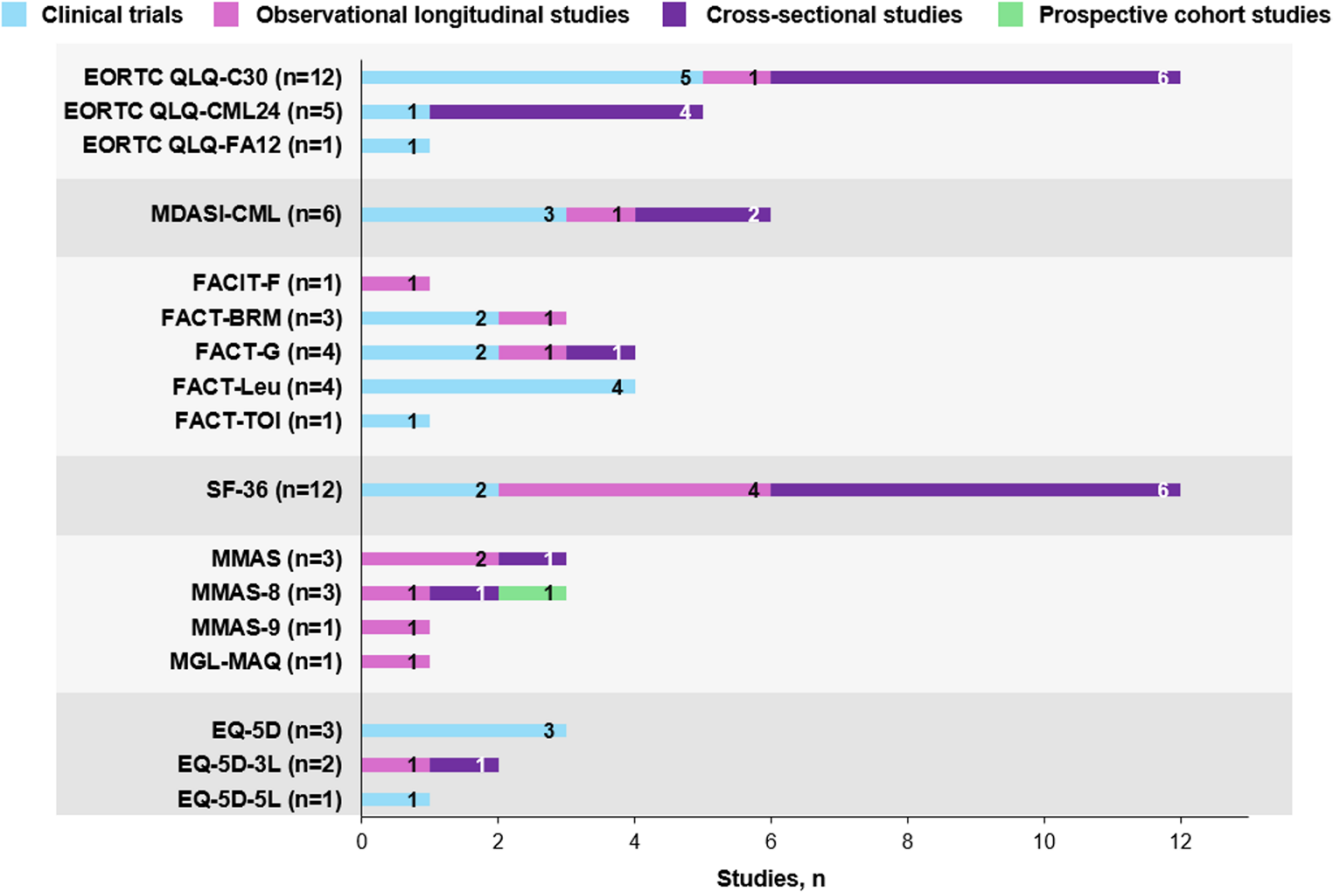
Commonly used PROMs in 1L CML studies. 1L, first line; 3L, 3-level; 5L, 5-level; BRM, biologic response modifiers; CML, chronic myeloid leukemia; EORTC, European Organisation for Research and Treatment of Cancer; EQ-5D, European Quality of Life 5 Dimensions; FA12, Fatigue questionnaire 12 items; FACIT-F, Functional Assessment of Chronic Illness Therapy Measurement System-Fatigue; FACT, Functional Assessment of Cancer Therapy; G, general; Leu, leukemia; MDASI, MD Anderson Symptom Inventory; MGL-MAQ, Morisky, Green, and Levine Medication Adherence Questionnaire; MMAS, Morisky Medication Adherence Scale; PROM, patient-reported outcome measure; QLQ-C30, Quality of Life Questionnaire Core-30; SF-36, 36-Item Short Form Survey; TOI, Trial Outcome Index

Of the 115 unique studies reporting the most commonly reported PROMs, 45 were in the frontline setting only, including 28 observational studies (cross-sectional, *n* = 17; prospective, *n* = 10; retrospective, *n* = 1), 15 clinical trials, and 2 prospective cohort studies. The most frequently used PROMs in these 45 frontline setting–only studies were EORTC QLQ and its variations (18 studies), FACT/FACIT (13 studies), and SF-36 (12 studies) (Fig. 3). Of these 45 studies, PROMIS was used in 1 frontline setting study, and the Patient-Reported Outcomes Version of the Common Terminology Criteria for Adverse Events (PRO-CTCAE) was not used. Table 2 highlights commonly used PROMs in the frontline setting, their areas of focus, and additional details. Hence, PROMIS and PRO-CTCAE are not shown in Fig. 3 or Table 2. Of the 36 studies reporting PRO data with study centers exclusively in the US, 15 were observational studies (cross-sectional, *n* = 5; prospective, *n* = 4; retrospective, *n* = 6), 12 were clinical trials, and 9 were neither observational nor clinical. Of the 36 US-only studies, 8 used the MDASI-CML, 5 used PROMIS, and 4 used SF-36. PRO-CTCAE was reported in 1 study, which was in the frontline setting.

**Table 2:** Commonly used PROMs in CML studies in the 1L setting

PROM	No. of studies	Type	Target population	Assessment areas	Recall period	No. of items	Time to completion	Available languages
**EORTC QLQ-C30 **[34, 47, 48]	12	Oncology specific	All cancer	Global health status Function • Physical • Role • Emotional • Cognitive • Social Symptoms • Fatigue • Nausea and vomiting • Pain • Dyspnea • Insomnia • Appetite loss • Constipation • Diarrhea Financial difficulties	7 days except for physical functioning (no recall period)	30	4 min (mobile)	120+
**EORTC QLQ-CML24** [49]	5	CML specific	CML	Symptom burden Impact on daily life Worry/mood Body image Satisfaction with care Social life	7 days	24	NA	NA
**EORTC QLQ-FA12** [50]	1	Oncology specific	All cancer	Fatigue (physical vs. emotional vs. cognitive)	7 days	12	NA	24
**EQ-5D** [51–54]	3	Generic	All conditions	Mobility Self-care Usual activities Pain/discomfort Anxiety/depression	Same day	6	2–5 min	> 150
**EQ-5D-3L** [51, 52, 55]	2	Generic	All conditions	Mobility Self-care Usual activities Pain/discomfort Anxiety/depression	Same day	6	2–5 min	> 150
**EQ-5D- 5L** [51, 53, 56]	1	Generic	All conditions	Mobility Self-care Usual activities Pain/discomfort Anxiety/depression	Same day	6	2–5 min	> 150
**FACIT-F** [24]	1	Generic	Chronic illness	Fatigue	7 days	13	< 5 min	67
**FACT-BRM** [57]	3	Oncology specific	All cancer	Physical Social Emotional Functional BRM-physical BRM-mental	7 days	40	10–15 min	27
**FACT-G** [58]	4	Oncology specific	All cancer	Physical Social Emotional Functional	7 days	27	5–10 min	75
**FACT-Leu** [59]	4	Leukemia specific	Leukemia	Physical Social Emotional Functional Leukemia	7 days	44	10–15 min	53
**FACT-TOI** [60]	1	Generic	All conditions	Physical Functional + Condition-specific scale	Variable	Variable	Variable	NA
**MDASI-CML** [37, 40]	6	CML specific	CML	Core MDASI symptoms • Pain • Fatigue • Nausea • Disturbed sleep • Distress • Shortness of breath • Difficulty remembering • Lack of appetite • Drowsiness • Dry mouth CML symptoms • Diarrhea • Swelling • Rash/skin change • Muscle soreness/cramping • Bruising/bleeding easily • Malaise • Headache MDASI interference • Relations with other people • Enjoyment of life • Mood • Walking • Activity • Work (including housework)	24 h	19	5 min	26
**MGL-MAQ** [61]	1	Generic	All conditions	Adherence	Unspecified	4	NA	NA
**MMAS** [62]	3	Generic	All conditions	Adherence	Unspecified or question specific	4	NA	35
**MMAS-8** [63]	3	Generic	All conditions	Adherence	Unspecified or question specific	8	NA	74
**MMAS-9** [64]	1	Generic	All conditions	Adherence	NA	9	NA	NA
**SF-36** [43, 65–67]	12	Generic	All conditions	Physical functioning Role physical Role emotional General health Social functioning Mental health Vitality Bodily pain	4 weeks/1 week/24 h	36	5–10 min	22

1L, first line; 3L, 3-level; 5L, 5-level; BRM, biologic response modifiers; CML, chronic myeloid leukemia; EORTC, European Organisation for Research and Treatment of Cancer; EQ-5D, European Quality of Life 5 Dimensions; FA12, Fatigue questionnaire 12 items; FACIT-F, Functional Assessment of Chronic Illness Therapy Measurement System-Fatigue; FACT, Functional Assessment of Cancer Therapy; G, general; Leu, leukemia; MDASI, MD Anderson Symptom Inventory; MGL-MAQ, Morisky, Green, and Levine Medication Adherence Questionnaire; MMAS, Morisky Medication Adherence Scale; NA, not applicable; PRO, patient-reported outcome; PRO-CTCAE, Patient-Reported Outcomes Version of the Common Terminology Criteria for Adverse Events; PROMIS, Patient-Reported Outcomes Measurement Information System; QLQ-C30, Quality of Life Questionnaire Core-30; SF-36, 36-Item Short Form Survey; TOI, Trial Outcome Index

## Discussion

The results of our scoping literature review yielded 6337 records of CML studies that used PROMs between January 2001 and September 2023, which was refined to 208 studies, based on preset inclusion and exclusion criteria. From these studies, the most commonly reported PROMs appearing in ≥ 5% of the studies were identified and included 92 unique PROMs. Of these 92 PROMs, EORTC QLQ-C30, SF-36, EORTC QLQ-CML24, FACT-Leu, and MDASI-CML were the 5 most commonly reported PROMs in studies across all lines, and EORTC measures, SF-36, and FACT/FACIT were most frequently reported in CML studies in the frontline setting. Most of these 92 PROMs were generic (67%), while some were specific to oncology (21%) and CML/leukemia/hematology (12%). This trend was consistently seen across all 208 CML studies and across the 81 studies in the frontline setting. However, across the 54 studies with ≥ 1 US study center, most PROMs were either generic or specific to CML/leukemia/hematology, while some were oncology specific.

Overall, our results underscore a lack of consensus on PROMs used in CML studies across lines of therapy and in frontline therapy. A recent real-world systematic review by Smit et al. concluded that none the 6 identified PROMs that assessed symptoms in patients with CML (EORTC QLQ-CML24, EORTC QLQ-C30, EORTC symptom set, FACT-Leu, a generic Chinese questionnaire, HM-PRO, and MDASI-CML) were sufficient for overall content validity [20]. Additionally, 5 of these PROMs (all except the generic Chinese questionnaire) were inconsistent due to not being evaluated by professionals post development, involving few patients with CML, or missing symptoms highly relevant to CML [20]. Smit et al. concluded that while new, validated CML-specific PROMs are needed, this effort must be guided by an understanding of patient preferences to ensure the tools are practical and meaningful in real-world settings [20].

According to our scoping literature search, the most used generic PROMs (reported in ≥ 3 studies) in CML in the frontline setting were SF-36, EQ-5D, MMAS, and MMAS-8. The MMAS and its variants assess medication adherence, not health-related QOL (HRQOL); moreover, they have been retracted [21]. The EQ-5D is available in the most languages, making it globally versatile, although it is considered most useful for cost-effectiveness research rather than as a comprehensive measure of HRQOL [12, 22]. It lacks sensitivity for measurement of HRQOL, and, with its same-day recall period, symptoms that wax and wane in diseases considered mild or asymptomatic may be missed [22]. Fatigue is a common adverse effect of TKIs [23], and the FACIT-F is fatigue specific; however, it needs to be used in conjunction other PROMs that can assess other aspects of QOL [24]. A prospective, longitudinal HRQOL study in patients with CML described the SF-36 as a well-established generic HRQOL measure but noted that it may not be sensitive enough to detect QOL changes in the CML population as it is not disease specific [25]. Notably, the SF-36 does not include gastrointestinal symptoms, a common adverse effect of many TKIs [26].

Oncology-specific measures focus on symptoms that are commonly experienced because of cancer and anticancer medications. Our results showed EORTC QLQ-C30, FACT-BRM, and Functional Assessment of Cancer Therapy-General (FACT-G) to be the most used oncology-specific PROMs (reported in ≥ 3 studies) in CML in the frontline setting. Other studies have identified EORTC measures as the most extensively used PROMs in cancer clinical trials and clinical practice [27–30]. Qualitative interviews from patients with cancer (in Europe and US) confirmed that concepts included in EORTC QLQ-C30 are relevant across cancer types and disease stages and are widely understood across language versions, hence establishing good content validity of EORTC QLQ-C30 [31]. A cross-sectional study in Kenyan patients with cancer also demonstrated the reliability and cross-cultural validity of EORTC QLQ-C30 for measuring QOL [32]. A limitation in scoring of EORTC measures is that thresholds for clinical importance need to be established to help healthcare professionals correctly identify and interpret changes in scores that are meaningful for patients [33]. One unique aspect of the EORTC QLQ-C30 is an assessment of financial difficulties, which may be a common experience in this patient population but is rarely included in PROMs, which are otherwise focused on symptoms and functioning [34].

A pilot randomized trial of the first cognitive behavioral intervention for TKI-related fatigue in CML used the FACT-G due to its established reliability, validity, and sensitivity to change in patients with cancer [23]. FACT-BRM was used in a QOL study with imatinib, and although it is designed to assess QOL in patients taking BRM, it was used because of its translation into other languages, validity, and coverage of a wide range of major HRQOL areas [35]. However, authors of another QOL study with imatinib noted that FACT-G, the first part of FACT-BRM, has been translated into other languages, but the subsequent parts of the questionnaire, BRM-physical and BRM-mental, have not been translated to Urdu, in particular, which limited data collection in their study, which was conducted in Pakistan [36].

CML-specific PROMs are designed to evaluate symptoms frequently experienced in this specific patient population. The most common CML-specific measures (reported in ≥ 3 studies) in the frontline setting in our scoping literature search were EORTC QLQ-CML24, MDASI-CML, and FACT-Leu. The MDASI-CML is a CML-specific PROM that is brief, validated, and designed for patients with CML. It comprises 20 core and CML-symptom specific items that assess symptoms that are particularly relevant to patients with CML and 6 interference items that assess how symptoms impact daily life [37, 38]. After completion of a phase 2 exploratory study assessing the effect of TKI switching on the AE profile in patients with low-grade toxicities, the MDASI-CML module was validated, and headache was added as a CML-specific item [39]. Each symptom in MDASI-CML is represented by a single question, thus limiting its sensitivity. Moreover, the recall period is 24 h, which can lead to missed symptoms that vary over longer time periods. It is available in fewer languages in comparison to many of the other PROMs, limiting its practical application in some cases [40].

The more recently developed, high-quality, generic PROMs, PROMIS and PRO-CTCAE, were reported in ≤ 5 studies in our results. While both PROMIS and PRO-CTCAE are highly customizable to the study context, they have some differences [41, 42]. The PRO-CTCAE is customizable and comprehensive, with 124 items representing amount, presence/absence, frequency, severity, and interference of 78 different toxicities. It is validated in 60 languages and has a recall period of 7 days [41]. However, the PRO-CTCAE provides descriptive reports of symptomatic toxicities at their worst and does not produce composite scores that can be used for interpretation [41]. Importantly, it includes items on attention and memory and sexual health, which are not captured by the commonly used PROMs, including EORTC QLQ-C30 and SF-36 [41, 43–45]. PROMIS was developed using advanced qualitative and psychometric methods, with customizable measures covering 70 domains of symptoms and functioning in the areas of pain, fatigue, and physical, mental/emotional, and social health [42]. Many of the measures use a 7-day recall period, and scores are normalized to the general population to facilitate interpretation of severity [42, 46]. Both PROMIS and PRO-CTCAE have extensive item libraries that can provide a comprehensive approach for evaluating patient outcomes [41, 42]. Hence, both require careful and reasonable curation to limit patient burden. Also, the highly customizable nature can be difficult to navigate for those implementing these generic tools compared with a CML-specific measure [46]. Our results showing limited use of these PROMs in CML studies in the frontline setting likely reflect both their more recent development as well as the complexities associated with the measure/item selection required for using PROMIS and PRO-CTCAE.

## Conclusion

Our scoping literature review demonstrated that a wide range of PROMs have been used to evaluate HRQOL in patients with CML in the frontline setting, including generic, oncology-specific, and CML-specific measures. While PROM heterogeneity makes cross-study comparability challenging, a general call for standardization is overly simplistic. Patients with CML who are treated with a TKI experience multiple and diverse impacts on HRQOL, and fit-for-purpose PROM selection depends on the study context, geographical region, accessibility, cost, time, language and literacy, and logistics of administration. Moreover, since CML is a chronic disease and TKIs are typically used as lifelong treatments, there is a need for obtaining information from patients regarding the impact on HRQOL throughout the continuum of care. An approach that balances fit-for-purpose measurement and comparability is needed. Many high-quality PROMs are available and appropriate for use in CML studies. A more comprehensive understanding of HRQOL will help optimize selection of patient-centered frontline treatment in CML. 

##  Key References

- Schoenbeck KL, Flynn KE. Health-related quality of life of patients with chronic myeloid leukemia as measured by patient-reported outcomes: current state and future directions.Curr Hematol Malig Rep. 2021;16(6):491-499.
  - The article provides an overview of how PROs have been used to assess HRQOL in patients with CML and highlights the limitations in existing PROs, identifying areas where further research is required.
- Shacham Abulafia A, Shemesh S, Rosenmann L, et al. Health-related quality of life in patients with chronic myeloid leukemia treated with first- versus second-generation tyrosine kinase inhibitors. J Clin Med. 2020;9(11):3417.
  - The article critically reviews the impact of lifelong TKI therapy on HRQOL, synthesizing evidence from multiple studies that used validated PRO tools. The authors highlight lack of longitudinal data and underrepresentation of certain patient subgroups as unmet needs in PRO research.
- Weldring T, Smith SM. Patient-reported outcomes (PROs) and patient-reported outcome measures (PROMs). Health Serv Insights. 2013;6:61-68.
  - The article provides a foundational understanding of how PROs and PROMs are used to assess HRQOL and offers insights into the methodological considerations of PROM development. The authors highlight the need for cultural adaptation and cost-effectiveness in the development of these instruments.
- Bull C, Teede H, Watson D, Callander EJ. Selecting and implementing patient-reported outcome and experience measures to assess health system performance. JAMA Health Forum. 2022;3(4):e220326.
  - The article provides a comprehensive guide to the selection and implementation of PROMs and describes key psychometric properties, such as validity, reliability, and responsiveness. The article also outlines key barriers to PROM implementation in the healthcare system.
- Smit Y, Metsemakers S, Janssen J, et al. Measuring chronic myeloid leukaemia TKI-related toxic effects in the real world: a systematic review and critical assessment of content validity of patient-reported outcome measures. Lancet Haematol. 2023;10(10):e849-e859.
  - The article evaluates 6 PROMs for assessing TKI-related toxic effects in patients with CML and concluded that none demonstrated sufficient content validity. Authors highlight the need for a validated and patient-centered PROM to monitor and manage TKI-related toxicities.
- Giesinger JM, Efficace F, Aaronson N, et al. Past and current practice of patient-reported outcome measurement in randomized cancer clinical trials: a systematic review. Value Health. 2021;24(4):585-591.
  - This article provides data on the historical and current use of PRO measures in randomized controlled trials across various cancer types.
- Smith AB, Cocks K, Parry D, Taylor M. Reporting of health-related quality of life (HRQOL) data in oncology trials: a comparison of the European Organization for Research and Treatment of Cancer Quality of Life (EORTC QLQ-C30) and the Functional Assessment of Cancer Therapy-General (FACT-G). Qual Life Res. 2014;23(3):971-976.
  - This article examines the implementation of PRO instruments in cancer trials, compares available tools, and evaluates the quality of HRQOL data reporting.
- Wintner LM, Sztankay M, Aaronson N, et al. The use of EORTC measures in daily clinical practice-A synopsis of a newly developed manual. Eur J Cancer. 2016;68:73-81.
  - This article examines the challenges of implementing PRO measures in clinical practice for patients with cancer. Authors provide a synopsis of newly developed tools and discuss practical considerations related to instrument selection, timing, scoring, and ethical issues.
- Pilz MJ, Thurner AMM, Storz LM, Krepper D, Giesinger JM. The current use and application of thresholds for clinical importance of the EORTC QLQ-C30, the EORTC CAT core and the EORTC QLQ-C15-PAL- a systematic scoping review. Health Qual Life Outcomes. 2025;23(1):55.
  - This article systematically reviews the use and application of thresholds for clinical importance in PROs and PROMs within oncology.
- Unnikrishnan R, Veeraiah S, Ganesan P. Symptom burden and quality of life issues among patients of chronic myeloid leukemia on long-term imatinib therapy. Indian J Med Paediatr Oncol. 2017;38(2):165-168.
  - This article evaluates the impact of symptom burden on QOL in patients with CML, providing insight into the use of PROs and PROMs in this population. Authors also validate the use of indigenous QOL tools.

## Supplementary Information

Below is the link to the electronic supplementary material. (DOCX 52.0 KB)

Supplementary Material 1 (PDF 1.50 MB)

## Data Availability

All data are available upon reasonable request from the corresponding author.
