# Supplementary material for: Use of Patient-Reported Outcome Measures in Clinical Studies of Chronic Myeloid Leukemia: A Scoping Literature Review
Source: Curr Hematol Malig Rep. 2025 Oct 15;20(1):15. doi: 10.1007/s11899-025-00755-0 (PMC12528340; doi:10.1007/s11899-025-00755-0)
Supplement: Supplementary file 1 — Supplementary Material 1 (PDF 1.50 MB) [file 11899_2025_755_MOESM1_ESM.docx]

**SUPPLEMENTARY APPENDIX**

**SUPPLEMENTARY TABLES**

**Table S1: Embase and MEDLINE search strategy for clinical trials using Embase.com.**

| **No.** | **Query** |
| --- | --- |
| 1 | 'chronic myeloid leukemia'/syn |
| 2 | 'chronic myelogenous leukemia':ab,ti OR 'chronic myelogenous leukaemia':ab,ti OR 'chronic myeloid leukaemia':ab,ti OR 'chronic myeloid leukemia':ab,ti OR 'cml':ab,ti OR 'cml-cp':ab,ti |
| 3 | 'chronic myel*' NEAR/3 leuk?emia |
| 4 | #1 OR #2 OR #3 |
| 5 | 'quality adjusted life year'/de OR 'quality of life index'/de OR 'short form 12'/de OR 'short form 20'/de OR 'short form 36'/de OR 'short form 8'/de OR 'sickness impact profile'/de OR 'quality of life':ab,ti OR 'sickness impact profile':ti,ab OR 'disability adjusted life':ti,ab OR qal*:ti,ab OR qtime*:ti,ab OR qwb*:ti,ab OR daly*:ti,ab OR euroqol*:ti,ab OR eq5d*:ti,ab OR 'eq5*':ti,ab OR 'eq 5d':ti,ab OR qol*:ti,ab OR hql*:ti,ab OR hqol*:ti,ab OR 'h qol*':ti,ab OR hrqol*:ti,ab OR 'hr qol*':ti,ab OR 'health utility*':ti,ab OR 'utility score*':ti,ab OR disutilit*:ti,ab OR 'utility value*':ti,ab OR hui:ti,ab OR hui1:ti,ab OR hui2:ti,ab OR hui3:ti,ab OR hye:ti,ab OR hyes:ti,ab OR 'discrete choice*':ti,ab OR rosser:ti,ab OR 'willingness to pay':ti,ab OR 'time tradeoff':ti,ab OR 'time trade off':ti,ab OR tto:ti,ab OR 'standard gamble*':ti,ab OR sf36*:ti,ab OR 'sf 36*':ti,ab OR 'short form 36*':ti,ab OR 'shortform 36*':ti,ab OR shortform36*:ti,ab OR sf20:ti,ab OR 'sf 20':ti,ab OR 'short form 20':ti,ab OR 'shortform 20':ti,ab OR shortform20:ti,ab OR sf12*:ti,ab OR 'sf 12*':ti,ab OR 'short form 12*':ti,ab OR 'shortform 12*':ti,ab OR sf8*:ti,ab OR shortform8*:ti,ab OR sf6*:ti,ab OR shortform6*:ti,ab OR 'mfsi-sf' OR 'rankin scale'/exp OR 'sis-16' OR 'caregiver burden'/de OR 'caregivers'/exp OR caregiv*:ti,ab OR nurs*:ti,ab OR 'informal care':ti,ab OR 'home care':ti,ab OR 'nursing home':ti,ab OR burden:ti,ab OR strain:ti,ab OR stress:ti,ab OR distress:ti,ab OR suffer*:ti,ab OR overload*:ti,ab |
| 6 | (('patient reported outcome*' OR 'self reported outcome*' OR 'quality of life'/exp OR 'quality of life' OR 'patient'/exp OR patient) AND ('satisfaction'/exp OR satisfaction) OR 'activities of daily living'/exp OR 'activities of daily living' OR 'life qualit*' OR 'activities of daily life' OR 'daily living activities' OR 'functional assessment'/exp OR 'functional assessment' OR 'pain measurement'/exp OR 'pain measurement' OR qol OR 'hrql'/exp OR hrql OR hrqol OR 'outcome assessment (health care)'/exp OR 'outcome assessment (health care)' OR 'health status'/exp OR 'health status' OR questionnaire* OR 'quality of life'/exp OR 'quality of life' OR 'questionnaires'/exp OR questionnaires OR (patient* AND adj2 AND reported) OR 'self'/exp OR self) AND report* OR 'patient* experience*' OR prom*1 OR 'severity of illness index'/exp OR 'severity of illness index' OR 'health utility' OR 'health status'/exp OR 'health status' OR 'psychosocial'/exp OR psychosocial OR 'patient satisfaction'/exp OR 'patient satisfaction' OR (outcome* NEAR/5 expectation*) OR (outcome* NEAR/5 satisfaction) OR (outcome* NEAR/5 (satisfaction OR satisfied)) OR (symptom* AND adj5 AND (improv* OR change* OR deteriorat*)) OR (patient* NEAR/5 priorit*) OR 'scale'/exp OR scale OR scales OR 'satisfaction'/exp OR satisfaction OR psychometric OR 'health-related quality of life'/exp OR 'health-related quality of life' OR hqol |
| 7 | #5 OR #6 |
| 8 | ('clinical trial'/exp OR 'randomization'/de OR 'controlled study'/de OR 'comparative study'/de OR 'single blind procedure'/de OR 'double blind procedure'/de OR 'crossover procedure'/de OR 'placebo'/de OR 'clinical trial' OR 'clinical trials' OR 'controlled clinical trial' OR 'controlled clinical trials' OR 'randomised controlled trial' OR 'randomised controlled trials' OR 'randomisation' OR 'randomization' OR rct OR 'random allocation' OR 'randomly allocated' OR 'allocated randomly' OR placebo* OR 'prospective study'/de OR (allocated NEAR/2 random) OR (random* NEAR/1 assign*) OR random* OR ((single OR double OR triple OR treble) NEAR/1 (blind* OR mask*))) NOT ('case study'/de OR 'case report' OR 'abstract report'/de OR 'letter'/de) |
| 9 | #4 AND #7 AND #8 |
| 10 | #9 AND [humans]/lim AND [english]/lim |
| 11 | #9 AND [humans]/lim AND [english]/lim AND [2001-2021]/py |
| 11.1 | #10 AND [29-07-2021]/sd |
| 12 | #9 AND [humans]/lim AND [english]/lim AND [2001-2021]/py AND ([article]/lim OR [article in press]/lim) |

**Table S2: Embase and MEDLINE search strategy for observational studies using Embase.com.**

| **No.** | **Query** |
| --- | --- |
| 1 | 'chronic myeloid leukemia'/syn |
| 2 | 'chronic myelogenous leukemia':ab,ti OR 'chronic myelogenous leukaemia':ab,ti OR 'chronic myeloid leukaemia':ab,ti OR 'chronic myeloid leukemia':ab,ti OR 'cml':ab,ti OR 'cml-cp':ab,ti |
| 3 | 'chronic myel*' NEAR/3 leuk?emia |
| 4 | #1 OR #2 OR #3 |
| 5 | 'quality adjusted life year'/de OR 'quality of life index'/de OR 'short form 12'/de OR 'short form 20'/de OR 'short form 36'/de OR 'short form 8'/de OR 'sickness impact profile'/de OR 'quality of life':ab,ti OR 'sickness impact profile':ti,ab OR 'disability adjusted life':ti,ab OR qal*:ti,ab OR qtime*:ti,ab OR qwb*:ti,ab OR daly*:ti,ab OR euroqol*:ti,ab OR eq5d*:ti,ab OR 'eq5*':ti,ab OR 'eq 5d':ti,ab OR qol*:ti,ab OR hql*:ti,ab OR hqol*:ti,ab OR 'h qol*':ti,ab OR hrqol*:ti,ab OR 'hr qol*':ti,ab OR 'health utility*':ti,ab OR 'utility score*':ti,ab OR disutilit*:ti,ab OR 'utility value*':ti,ab OR hui:ti,ab OR hui1:ti,ab OR hui2:ti,ab OR hui3:ti,ab OR hye:ti,ab OR hyes:ti,ab OR 'discrete choice*':ti,ab OR rosser:ti,ab OR 'willingness to pay':ti,ab OR 'time tradeoff':ti,ab OR 'time trade off':ti,ab OR tto:ti,ab OR 'standard gamble*':ti,ab OR sf36*:ti,ab OR 'sf 36*':ti,ab OR 'short form 36*':ti,ab OR 'shortform 36*':ti,ab OR shortform36*:ti,ab OR sf20:ti,ab OR 'sf 20':ti,ab OR 'short form 20':ti,ab OR 'shortform 20':ti,ab OR shortform20:ti,ab OR sf12*:ti,ab OR 'sf 12*':ti,ab OR 'short form 12*':ti,ab OR 'shortform 12*':ti,ab OR sf8*:ti,ab OR shortform8*:ti,ab OR sf6*:ti,ab OR shortform6*:ti,ab OR 'mfsi-sf' OR 'rankin scale'/exp OR 'sis-16' OR 'caregiver burden'/de OR 'caregivers'/exp OR caregiv*:ti,ab OR nurs*:ti,ab OR 'informal care':ti,ab OR 'home care':ti,ab OR 'nursing home':ti,ab OR burden:ti,ab OR strain:ti,ab OR stress:ti,ab OR distress:ti,ab OR suffer*:ti,ab OR overload*:ti,ab |
| 6 | (('patient reported outcome*' OR 'self reported outcome*' OR 'quality of life'/exp OR 'quality of life' OR 'patient'/exp OR patient) AND ('satisfaction'/exp OR satisfaction) OR 'activities of daily living'/exp OR 'activities of daily living' OR 'life qualit*' OR 'activities of daily life' OR 'daily living activities' OR 'functional assessment'/exp OR 'functional assessment' OR 'pain measurement'/exp OR 'pain measurement' OR qol OR 'hrql'/exp OR hrql OR hrqol OR 'outcome assessment (health care)'/exp OR 'outcome assessment (health care)' OR 'health status'/exp OR 'health status' OR questionnaire* OR 'quality of life'/exp OR 'quality of life' OR 'questionnaires'/exp OR questionnaires OR (patient* AND adj2 AND reported) OR 'self'/exp OR self) AND report* OR 'patient* experience*' OR prom*1 OR 'severity of illness index'/exp OR 'severity of illness index' OR 'health utility' OR 'health status'/exp OR 'health status' OR 'psychosocial'/exp OR psychosocial OR 'patient satisfaction'/exp OR 'patient satisfaction' OR (outcome* NEAR/5 expectation*) OR (outcome* NEAR/5 satisfaction) OR (outcome* NEAR/5 (satisfaction OR satisfied)) OR (symptom* AND adj5 AND (improv* OR change* OR deteriorat*)) OR (patient* NEAR/5 priorit*) OR 'scale'/exp OR scale OR scales OR 'satisfaction'/exp OR satisfaction OR psychometric OR 'health-related quality of life'/exp OR 'health-related quality of life' OR hqol |
| 7 | #5 OR #6 |
| 8 | clinical study'/de OR 'case control study'/exp OR 'family study'/de OR 'longitudinal study'/de OR 'retrospective study'/de OR 'prospective study'/de OR 'cross sectional study'/de OR 'cohort analysis'/de OR ('follow up'/de AND cohort*:ti,ab) OR 'case control':ti,ab OR cohort*:ti,ab OR 'case series':ti,ab OR 'clinical series':ti,ab OR ((('follow up' OR observational OR uncontrolled OR 'non randomi?ed' OR nonrandomi?ed OR epidemiologic*) NEXT/1 (study OR studies)):ti,ab) OR ((longitudinal:ti,ab OR retrospective:ti,ab OR prospective:ti,ab OR 'cross sectional':ti,ab) AND (study:ti,ab OR studies:ti,ab OR review:ti,ab OR analys*:ti,ab OR cohort*:ti,ab)) OR 'systematic review' OR 'questionnaire'/exp OR 'health survey'/exp OR 'survey methodology'/exp OR 'database':ti,ab OR 'register*':ti,ab OR 'registry':ti,ab OR 'registries':ti,ab OR 'real world':ti,ab OR 'non interventional':ti,ab OR 'nis':ti,ab OR 'survey':ti,ab |
| 9 | #4 AND #7 AND #8 |
| 10 | #9 AND [humans]/lim AND [english]/lim |
| 11 | #9 AND [humans]/lim AND [english]/lim AND [2001-2021]/py |
| 11.1 | #10 AND [29-07-2021]/sd |
